# Supplementary material for: Job Satisfaction Level of Safety and Health Manager in Construction Industry: Pandemic Period
Source: Int J Environ Res Public Health. 2022 May 11;19(10):5858. doi: 10.3390/ijerph19105858 (PMC9141010; doi:10.3390/ijerph19105858)
Supplement: Supplementary file 1 [file ijerph-19-05858-s001.zip › ijerph-1692464-supplementary.pdf]

A Survey on the Job Satisfaction of Health Safety Managers in the Construction Industry

This study is to investigate the job satisfaction of health and safety managers in the construction industry and create data that can be used in the field to help them perform business management tasks.
To this end, we would like to understand what should be included in the practical guidelines, what health and safety tasks should be done in the construction industry, and job satisfaction, so please respond to the survey even if you are busy.
This survey is for health and safety managers in the construction industry who voluntarily agree to the survey, and the survey contents and personal matters are treated confidentially, and all the results are coded and used only for research, and personal information is strictly confidential. In addition, we inform you that you can reject the survey at any time, and there is no disadvantage even if you reject it.

I agree with the completion of this survey, and I heard enough explanation about the research before completing it.

Agree □

<Inquiry> Seoultech : Won Choi 010-8526-9856
Seoultech : Sang-joon Lee 010-3280-3077

Occupational Hygiene Lab, Seoul Nationl University of Science & Technology

I. The following questionnaire is about your general characteristics. Please mark it where applicable.

1. Sex
□ ① Male □ ② Female

2. Age
( )

3. Final academic background
□ ① Under high school graduate □ ② Junior college □ ③ College □ ④ Graduate school

4. Marital status
□ ① Single □ ② Married □ ③ Divorce/Bereavement/Separation

5. Current role and certificate
□ ① Health manager
□ Nurse □ Industrial hygiene management engineer/industry engineer
□ Atmospheric engineer/industry engineer □ Others ( )
□ ② Safety manager
□ Industial safety instructor □ Industrial safety engineer/industry engineer
□ Construction safety engineer/industry engineer □ Others ( )
□ ③ Others ( )

6. Position
□ ① Head nanager □ ② Assistant manager □ ③ Chief/Staff

7. Nunber of years in Construction industry
(years month)

8. Number of years in current position
(years month)

9. Department
□ ① Safety/Health □ ② General affairs/Management □ ③ Nursing care
□ ④ Facility □ ⑤ Others ( )

10. Form of employment
□ ① Permanent □ ② Contract □ ③ Assigned □ ④ Part-time □ ⑤ Others ( )

11. Average working hours per week
□ ① 40 or less □ ② 41-48 or less □ ③ 49-56 or less □ ④ More than 57

12. Is there and opportunity for you company to promote itself as a health (safety) manager?
□ ① Yes □ ② Yes, but limited □ ③ No

13. Have you ever had an accident (physical injury) while on duty?
□ ① Yes □ ② No

II. The following questionnaire is about your work-related characteristics. Please mark it where applicable.

1. Where is the location of the construction site you are currently working at?
<   City/Province> <   Distric/County>

2. What is the size of the construction site you are currently working on?
□ ① Less than 100 household □ ② 100~500 household □ ③ 500~1000 household
□ ④ 1000~2000 household □ ⑤ More than 2000 household

3. How many workers are working at the construction site you are currently working on?
□ ① Less than 50 □ ② 50~100 □ ③ 100~300
□ ④ 300~500 □ ⑤ 500~1000 □ ⑥ More than 1000

4. What kind of construction site you are currently working on?
□① Building contruction (apartment/hospital/building/factory)
□② Civil engineering work (roal/port/bridge/sewage treatment)
□③ Plant (chemical/refinery/gas/power plant/encvironmnet plant)
□④ Railroad/track

5. What types of health (safety) management do you have?
□ ① Full-time □ ② Adjunct □ ③ Self-appointment & agency □ ④ Agency

6. How many safety/health managers in construction site you are currently working on?
1) Safety manager(   ) 2) Health manager(   )

7. What is the type of health care room (medical room) installed in the workplace?
□ ① Independent space □ ② Combined with other workspaces □ ③ No health care room

8. Are there any labor unions in the workplace?
□ ① Yes □ ② No

9. How about the establishment and operation of the Occupational Safety and Health Committee?
□ ① Not established □ ② Replaced by a labor-management council
□ ③ Established, but not operated □ ④ Established and operated regulary

10. How many other tasks do you do other than the safety (health) manager's own?
□ ① Don't do □ ② 25% of own work □ ③ 50% of own work
□ ④ 75% of own work □ ⑤ Always do

11. If you are a safety manager, what do you actually do? (respond only if you are a safety manager)

Question		Do	Donot
1. Safety management	1) Selection of qualified products when purchasing machines and apparatuses subject to mandatory safety certification, machines and apparatus subject to autonomous safety verification, etc		
	2) Establishing and implementing a safety education plan		
	3) Suggestions for workplace inspection, guidance, and safety measures		
2. Others	1) Suggestions for measures taken against workers who violate safety-related matters among orders under the Act or safety and health management regulations and employment rules		
	2) Investigation of the causes of industrial accidents and establishment of countermeasures		
	3) Management of industrial safety-related documents such as industrial accident statistics		
	4) Work related to industrial accident compensation (Preparation of Application for Industrial Accident Treatment, etc.)		
	5) Provision of industrial safety data/information		
	6) Work related to inspection of Ministry of Employment and Labor		
● If you have any other work, please write it down.			

12. If you are a health manager, what do you actually do? (respond only if you are a health manager)

Question		Do	Do not
1. Workplace environment management	1) Workplace environment measurement and follow-up management for it's results		
	2) Publication or storage of material safety data sheets		
	3) Inspection of ventilation systems and local exhaust systems		
	4) Suggestions for workplace inspection, guidance, and safety measures		
	5) Engineering improvement of work methods		
	6) Selection of qualified products when purchasing protective equipment and wearing guidance		
2. Health care for workers	1) Establishing and implementing a health education plan		
	2) Health counseling		
	3) Operation of health promotion program<br>(No smoking, stop drinking, exercising, job stress management, etc.)		
	4) Health examination and follow-up management for it's results		
	5) General disease management (general medicine supply, first aid, etc.)		
	6) Preventive management of cerebrovascular disease		
	7) Preventive management of musculoskeletal diseases		
3. Others	1) Suggestions for measures taken against workers who violate health-related matters among orders under the Act or safety and health management regulations and employment rules		
	2) Investigation of the cause of occupational diseases and establishment of countermeasures		
	3) Management of industrial accident workers (Return to work rehabilitation, etc.)		
	4) Management of industrial health-related documents such as industrial accident statistics		
	5) Work related to industrial accident compensation<br>(Preparation of Application for Industrial Accident Treatment, etc.)		
	6) Operating health management room (medical room), physical therapy room, etc		
	7) Work related to injection needle damage and stabbing accidents		
	8) Provision of industrial health data/information		
	9) Work related to inspection of Ministry of Employment and Labor		
● If you have any other work, please write it down.			

13. What do you think of the following when it comes to health and safety?

Question	Absolutely No	No	Normal	Yes	Strongly yes
1. The government's legal and institutional support is appropriate					
2. Business owners are highly aware of safety and health					
3. Workers engage in voluntary participation and cooperation					
4. The recognition and support of the senior department head is appropriate					
5. The cooperation of other departments in the workplace is high					
6. High coordination with other safety (health) managers in the workplace					
7. Financial support in the workplace is appropriate					
8. Require formal health and safety management activities from the safety (health) manager					
9. The connection with external organizations is smooth					
10. The salary level is appropriate					
11. The status of the workplace and working conditions are stable					
12. Job autonomy or job authority is free					
13. Administration and paperwork are appropriate					
14. Education to improve work skills is appropriate					
15. I have a lot of job stress					

III. The following questionnaire is about your job stress. Please mark it where applicable.				
Question	Absolutely No	No	Yes	Strongly yes
1. I'm always pressed for time because I have a lot of work	1	2	3	4
2. The workload has increased significantly	1	2	3	4
3. Sufficient rest is given during the work	4	3	2	1
4. have to do many things at the same time	1	2	3	4
5. My work requires creativity	4	3	2	1
6. I need a high level of skill or knowledge to perform my work	4	3	2	1
7. During work hours and in the process of performing work, I am given the authority to make decisions and can exert influence	4	3	2	1
8. I can adjust my workload and work schedule by myself	4	3	2	1
9. My boss helps me complete my work	4	3	2	1
10. My colleague helps me complete my work	4	3	2	1
11. There are people who understand that I am having a hard time at work	4	3	2	1
12. The future is uncertain because of the unstable situation at work	1	2	3	4
13. There have been or are expected to be undesirable changes in my working conditions or circumstances (e.g., restructuring)	1	2	3	4
14. The work evaluation and personnel system (promotion, departmental arrangement, etc.) are fair and reasonable	4	3	2	1
15. Support for personnel, space, facilities, equipment, and training necessary for performing works is well provided	4	3	2	1
16. There is no friction between our department and other departments and business cooperation works well	4	3	2	1
17. There are opportunities and channels to reflect my thoughts on work	4	3	2	1
18. Considering all my hard work and achievements, I am properly respected and trusted at work	4	3	2	1

19. Thinking that my situation will get better in the future makes me work without knowing it's hard.	4	3	2	1
20. I am given the opportunity to develop and demonstrate my abilities	4	3	2	1
21. The company dinner is uncomfortable	1	2	3	4
22. receive work instructions in an inconsistent	1	2	3	4
23. The atmosphere at work is authoritative and vertical	1	2	3	4
24. Be disadvantaged by sexual differences	1	2	3	4

IV. The following questionnaire is about your job satisfaction. Please mark it where applicable.					
Question	Absolutely No	No	Normal	Yes	Strongly yes
1) What I'm doing is fun and interesting	1	2	3	4	5
2) I'm satisfied with what I'm doing	1	2	3	4	5
3) What I'm doing makes me feel fulfilled	1	2	3	4	5
4) The workload of What I'm doing is moderate	1	2	3	4	5
5) What I'm doing is a job that is respected by others	1	2	3	4	5
6) I have a lot of discretion in my work	1	2	3	4	5
7) I maintain a friendly relationship with other departments in relation to my work	1	2	3	4	5
8) My job at work is stable	1	2	3	4	5
9) The job rewards me appropriately in proportion to my efforts and achievements	1	2	3	4	5
10) I am satisfied with the given workload	1	2	3	4	5
11) I am satisfied with the employee welfare system (facilities, childbirth, etc.)	1	2	3	4	5
12) I am satisfied with the salary level for the workload	1	2	3	4	5
13) The working environment and working conditions are better than other jobs	1	2	3	4	5
14) I am satisfied with the management of the company	1	2	3	4	5
15) I am satisfied with my boss's ability to perform his duties	1	2	3	4	5
16) I have a longing for a lifelong job	1	2	3	4	5

◎ Thank you for answering the survey ◎
